# Ethnic cultural influences on driving risk: a mixed-methods analysis of Cantonese, Hakka, and Hoklo drivers in China

**DOI:** 10.3389/fpubh.2026.1728417

**Published:** 2026-03-13

**Authors:** Guangnan Zhang, Mingqin Chen

**Affiliations:** Center for Studies of Hong Kong, Macao and Pearl River Delta, Institute of Guangdong, Hong Kong and Macao Development Studies, Sun Yat-sen University, Guangzhou, China

**Keywords:** driving behavior, ethnic culture, qualitative comparative analysis, risk factor, traffic accident risk

## Abstract

**Introduction:**

With the acceleration of globalization and inter-regional traffic, road accidents have become a leading cause of death worldwide. Drivers from outside ethnic cultural areas face greater traffic safety challenges compared to local drivers. While ethnic culture significantly shapes driving habits, its role in traffic safety remains under-researched, particularly within unified legal and infrastructural frameworks. This study addresses this gap by investigating the risk patterns of Cantonese, Hakka, and Hoklo drivers in Guangdong, China.

**Methods:**

Using a mixed-methods approach incorporating the induced exposure method to analyze traffic accident data from 2006 to 2010, we evaluate the interplay between individual, vehicle, and cultural factors. Specifically, we applied logistic regression to determine the odds of fault and Qualitative Comparative Analysis (QCA) to identify configurational risk pathways.

**Results:**

The logistic model revealed significant behavioral differences among the three ethnic groups, with factors such as occupation, vehicle use, and weather impacting the groups differently. Both regression analysis and QCA indicated that young drivers had a higher accident risk. Although gender, household registration, and occupations were not significant in the logistic models for Hakka and Hoklo groups, QCA showed these factors played crucial roles when combined with other elements.

**Discussion:**

This study uncovered multiple patterns in driving behavior across different ethnic cultures, enhancing the understanding of cultural influences on traffic accident risk. The findings reveal distinct cultural footprints in driving risks, offering a concrete basis for targeted, culturally-sensitive traffic interventions.

## 1 Introduction

While globalization has facilitated the inter-regional flow of resources and people, it has also intensified the discord between non-local drivers and regional cultural or legal frameworks (1, 2). As such, the risky behavior of drivers from different cultures has received extensive academic attention (26, 31–33). Due to factors, such as the ecological environment, local culture and traditions, economic context, and cultural exchange, drivers’ risk preferences in areas other than the region in which they live differ greatly depending on their ethnic backgrounds (3, 4), leading to significant differences in driving behavior, and ultimately leading to higher risks when driving in areas with different ethnic cultures. Empirical evidence supports these disparities: Hispanic drivers in the U.S., Black children in London, and Arabs in Israel all exhibit higher risks of violations or injuries compared to other ethnic groups within their respective regions (5–7).

Despite established ethnic disparities in traffic risks, existing research often adopts narrow perspectives—focusing either on regional economic development or isolated individual traits—thereby lacking a systematic framework that integrates driver, vehicle, road, and environmental factors. Furthermore, heavy reliance on aggregate macro-data often obscures individual-level nuances, potentially leading to confounding effects in cross-cultural comparisons (5). Conversely, micro-level studies utilizing questionnaire surveys frequently suffer from inherent sample bias, as they focus on self-reported tendencies rather than objective, accident-specific records. Furthermore, prior research has largely struggled to isolate the pure effect of ethnic culture on driving behavior, as these studies often fail to effectively control for confounding geographical and environmental variables. Although specific cultural traits—such as marine culture, gambling traditions, and religious beliefs—have been linked to economic risk preferences in corporate investment and innovation (34, 8), their direct implications for traffic accident risk remain significantly under-explored. Moreover, traditional categorizations based on nationality or race frequently overlook intra-national cultural diversity, thereby confounding the precise mechanisms through which ethnic sub-cultures influence driving behaviors. To address these gaps, this study utilizes the Chinese Ministry of Public Security’s Road Traffic Accident Database (2006–2010) to analyze ethnic cultural influences within a comprehensive driver-vehicle-road-environment framework. By integrating logistic regression with Qualitative Comparative Analysis (QCA), we systematically examine the complex risk configurations of Hakka and Hoklo drivers operating within Cantonese cultural regions.

The Cantonese, Hakka, and Hoklo sub-cultures within Guangdong Province were strategically selected for this investigation based on the following justifications. First, by restricting the sample to Guangdong, all participants operate within a singular institutional and legal environment. Unlike transnational studies—such as those comparing Malaysian drivers in Singapore—this localized approach effectively isolates cultural determinants by neutralizing the confounding effects of disparate traffic laws and national infrastructures. Second, as these groups are sub-sets of the broader Han ethnicity, this intra-ethnic focus offers a more granular perspective than traditional racial categorizations (e.g., African, Asian, or Hispanic). This design minimizes intra-racial heterogeneity, allowing for a more precise extraction of cultural risk factors. Third, these sub-cultures align strictly with three distinct linguistic lineages—Cantonese, Hakka, and Hoklo (identified by the Min dialect)—enabling the use of native dialect as a robust proxy for cultural background (35). This linguistic identification minimizes errors common in nationality-based or self-reported ethnic metrics.

This study has four main contributions: (1) By focusing on Cantonese, Hakka, and Hoklo sub-cultures within Guangdong, this study isolates ethnic cultural influences on risky driving behavior. Unlike cross-national or racial comparisons, our intra-regional approach effectively neutralizes the confounding effects of disparate legal systems, institutions, and national socio-economic policies. (2) Addressing the limitations of prior survey-based research—which often suffers from selection bias and omitted variable concerns—this study employs a comprehensive driver-vehicle-road-environment framework. This systematic approach allows for a more robust analysis of cultural risk factors across multiple operational dimensions. (3) To mitigate potential overfitting and variable dependency issues, this study integrates logistic regression with Qualitative Comparative Analysis (QCA). This mixed-methods design provides a configurational understanding of traffic safety as an open system, capturing complex interactions between cultural traits and environmental factors across micro, meso, and macro levels. (4) Finally, this investigation expands the scholarship on ethnic culture and traffic safety by validating linguistic-based identification methods and introducing novel analytical lenses to explore culturally-embedded driving behaviors.

The remainder of the paper is organized as follows: Section 2 reviews the past studies on culture and risk propensity, ethnic culture and traffic accident risk and analyses existing issues. Section 3 introduces the research data, model, and method, and proposes solutions to the issues raised in past literature. Section 4 presents the logistic regression model’s results, which analyze the differences in traffic accident risk between drivers of the three ethnic cultures in the Cantonese areas. Section 5 presents the QCA results, which investigates Hakka and Hoklo drivers’ risky driving behavior in the Cantonese areas. Section 6 discusses risk factors and risky driving behavior in other ethnic cultures’ areas. Finally, Section 7 presents the conclusions.

## 2 Literature review

With globalization and economic development, driving in regions of other ethnic cultures has become increasingly common. One example is that of EU drivers who can drive freely across EU countries (36).

From an economic perspective, culture acts as a fundamental determinant of utility-based preferences, leading to significant heterogeneity in risk-taking behavior across ethnic groups (3, 4). Global evidence suggests that these culturally ingrained preferences directly translate into varied propensities for risk across different populations. For instance, specific sub-cultures, such as those characterized by marine traditions or gambling norms, have been empirically linked to heightened risk engagement in corporate investments and financial stability (34, 8). These broad cultural patterns extend to road safety; for example, populations with lower patience or higher fatalistic beliefs—traits often clustered in specific geographic or ethnic regions—exhibit more dangerous behaviors on the road (3). Furthermore, variations in driving participation among immigrant groups underscore how cultural heritage continues to shape mobility choices in new environments (37). Since roads are the primary means of satisfying growing transportation demands (9), driving behavior has become one of the most common risk-taking behaviors.

While cultural factors are widely acknowledged to influence driving behavior, identifying specific cultural determinants remains challenging (5). Current research categorizes these influences into psychological and social dimensions, including drivers’ general attitudes, religious beliefs, regional identities, and the social resistance exhibited by marginalized groups (32, 33). Notably, internal belief systems—particularly fatalism—operate through similar psychological mechanisms to shape risk perception. Fatalistic individuals often attribute accidents to external, uncontrollable factors rather than personal driving errors, leading to a systematic disregard for safety measures and more dangerous road behaviors (38, 39, 10, 11). Consequently, these ingrained attitudes act as cultural filters that regulate how drivers internalize safety norms and respond to environmental hazards.

Research on non-local or foreign drivers suggests they face elevated accident risks, often attributed to a combination of language barriers, unfamiliar driving standards, and a lack of local environmental knowledge. Crucially, these drivers frequently retain the ingrained driving habits of their home regions, which may conflict with the requirements of a new driving environment. This safety disadvantage is further amplified by disparities in infrastructure, legal frameworks, and social systems between regions, often resulting in more severe injury outcomes for foreign drivers in collisions (13, 14). Applying an analytical framework of personal and environmental factors, Harootunian et al. (12) examined interstate driving risks in the U.S., finding that risk factors for out-of-state drivers lacked cross-state consistency. Similarly, Zhang et al. (2) demonstrated that even within the same ethnic group, drivers exhibited divergent behaviors when crossing borders between Guangdong, Hong Kong, and Macao. Interestingly, some scholars report contradictory findings, suggesting that foreign drivers may exhibit safer, more cautious behaviors as a compensatory response to unfamiliar cultural and social systems (40). These conflicting results indicate that increased traffic risk may not stem from geographical or administrative boundaries per se, but rather from the underlying ethnic cultural backgrounds of the drivers.

Research consistently indicates that marginalized ethnic minorities face disproportionately higher risks of traffic accidents (6, 7, 15). However, the underlying mechanisms—whether stemming from socioeconomic disparities or cultural friction—remain a subject of ongoing debate (5). Empirical evidence across diverse contexts—ranging from Hispanic drivers in the U.S. to Black children in London—highlights consistent disparities in violation rates and injury outcomes (6, 7, 15). These disparities are often linked to lower compliance with safety measures, such as seat belt usage, among minority groups. Notably, Elias et al. (5) theorized that social marginalization can foster a “culture of mistrust,” where perceived discrimination translates into a conscious disregard for traffic laws. While their study on Israeli Arabs provided a compelling conceptual framework, it lacked sufficient empirical data to fully validate the causal link between cultural resentment and accident risk.

Methodologically, existing literature bifurcates into macroscopic comprehensive models (16, 17) and microscopic questionnaire surveys (5). However, both approaches face significant hurdles: macro-level studies often focus on aggregate behavioral proportions (e.g., seat belt usage) without granular accident-specific data, while micro-level surveys frequently lack objective validation from actual traffic records (6). For instance, while (17) observed lower traffic mortality in developed Cantonese-dominant regions compared to Hakka and Hoklo areas, their analysis primarily attributed these disparities to institutional factors like injury prevention legislation and traffic calming measures, rather than the intrinsic cultural backgrounds of the drivers themselves. Furthermore, micro-level studies that focus on individual traits often fail to account for the complex interactions within a systematic driver-vehicle-road-environment framework, potentially overlooking key environmental moderators (6, 18).

In summary, while existing literature establishes a theoretical link between cultural diversity and traffic risk, empirical validations often conflate culture with confounding variables such as language barriers, geographical disparities, and infrastructure quality. This lack of isolation prevents a precise understanding of how ethnic culture, as a standalone factor, influences driving behavior. Furthermore, the heavy reliance on self-reported survey data, rather than objective accident records, introduces potential selection bias and ignores the complex interactions within a systematic driver-vehicle-road-environment framework. To address these limitations, this study utilizes objective data from the Chinese Road Traffic Accident Database (2006–2010) within the unique context of Guangdong Province. By focusing on Cantonese, Hakka, and Hoklo sub-cultures—who share a unified legal and infrastructural environment—we effectively isolate cultural effects from institutional and geographical noise. Based on this framework, we test the following hypotheses:

*H1*: Ethnic sub-cultures generate distinct driving risk patterns even when operating under a unified legal and infrastructural framework.

*H2*: Geographic-cultural adaptation (e.g., marine vs. mountain cultures) leads to divergent behavioral responses to environmental stressors such as bad weather.

*H3*: Cultural risk is configurational in nature, meaning certain individual traits only influence accident odds when combined with specific vehicle or occupational configurations.

## 3 Data and model

### 3.1 Data source and variable operationalization

Existing studies mainly categorized ethnic groups and cultures by nationality/region (such as Malaysians driving in Singapore or US drivers driving in other states), race (such as white or black), and descent (such as Asian and Hispanic), to study the differences in accident risk between ethnic cultures (5–7). However, these broad categorizations often fail to capture the granular nuances of ethnic culture. In culturally diverse nations like China, significant behavioral disparities exist within the same race or descent, which traditional metrics—such as nationality or racial grouping—cannot adequately isolate. Furthermore, nationality-based classification conflates cultural traits with institutional factors, such as disparate traffic laws or road rules (e.g., left-side versus right-side driving). By restricting this study to Guangdong Province, we effectively neutralize these institutional variables, ensuring a homogeneous legal and infrastructural baseline. Crucially, the three predominant sub-cultures in Guangdong—Cantonese, Hakka, and Hoklo—perfectly align with three distinct linguistic branches: Cantonese, Hakka, and Min dialect (the linguistic foundation of Hoklo culture). Consequently, this study utilizes native dialect as a rigorous proxy for ethnicity, facilitating a more precise identification of cultural background while minimizing the identification errors inherent in self-reported or administrative data.

There are several advantages to identifying the cultural background of ethnic groups through their native dialect: (1) Linguistically, Chinese dialects serve as the primary repositories of regional heritage; thus, a specific dialect is inherently and systematically aligned with its localized culture (41). Variations in phonology, lexicon, and syntax not only create communication barriers but also encapsulate deep-seated cultural heterogeneities (42). (2) Because linguistic structures are deeply embedded within social matrices, they exhibit long-term continuity and stability, reflecting evolutionary trajectories that are resistant to short-term institutional shifts (43). (3) Each dialect acts as a cognitive framework that shapes regional thinking and behavioral patterns (44, 45). Within multicultural regions, shared linguistic heritage fosters common social norms and codes of conduct. A salient example is the Hakka people’s profound linguistic identity, encapsulated in their ancestral motto: “Better to lose the land of ancestors than to lose the tongue of ancestors,” which serves to preserve their distinct cultural mores and internal cohesion (46). (4) Crucially, the three predominant sub-cultures in Guangdong—Cantonese, Hakka, and Hoklo—perfectly align with three distinct linguistic branches: Cantonese, Hakka, and Min (35). This precise correspondence allows for the spatial and demographic identification of approximately 60 million Cantonese, 20 million Hakka, and 18.95 million Hoklo residents, concentrated across specific river basins and coastal corridors (47).

This study’s dialect data was extracted from the “Dialects Attributable to Counties in China in 1986.” The book was systematically compiled by the team of Xu from Lingnan College of Sun Yat-sen University based on “The Great Dictionary of Chinese Dialects” (*The Dictionary*) (Edited by Xu and Ichiro, Zhonghua Book Company, 1999). *The Dictionary* divides Chinese dialects into dialectic regions, areas, and segments. It records the dialects of each county according to the 1986 administrative divisions. This table was compiled in full respect of the records in *The Dictionary* and matched with the administrative divisions and codes of the Ministry of Civil Affairs of 1986. However, *The Dictionary* only covered 92.2% of the county-level administrative units (2,615 out of 2,835). Therefore, although counties not covered by *The Dictionary* were also included in the table, there were no dialects attributed to them.

The traffic accident data were extracted from accident reports from the Ministry of Public Security’s Road Traffic Accident Database from 2006 to 2010. This database is the only formal and legitimate source officially published in China, and it is the most detailed and reliable source for research on Chinese road traffic safety. The data includes information on the characteristics of the parties involved in each accident, the number of casualties, vehicle characteristics, road conditions, time, and the environmental context (19). Although this study only used the traffic accidents in 21 cities in Guangdong Province from 2006 to 2010, given that the severity of road traffic accidents in Guangdong ranks first in the country and the province has the largest migrating population in China, the data remained representative and reasonably generalizable in analyzing developing countries’ traffic safety issues (30, 31). Moreover, all data were fully anonymized by the issuing authority before being accessed for research purposes, with no personal identifiers (such as names, ID numbers, or specific contact details) available to the authors. As the research involves solely the statistical analysis of pre-existing, de-identified administrative records and does not entail direct human intervention or clinical trials, it was conducted in accordance with the institutional ethical standards for secondary data analysis.

To address the absence of vehicle-miles-traveled (VMT) data, we utilized the induced exposure method, a robust quasi-experimental design widely validated in traffic safety scholarship (20). This method identifies relative risk by comparing “at-fault” drivers with a control group of “innocent” (non-fault) drivers involved in the same multi-vehicle collisions. The core theoretical premise, as established by Stamatiadis and Deacon (20), is that the characteristics of not-at-fault drivers (the “innocent victims”) provide a random sampling of the exposure distribution, representing the overall driving population at risk. Specifically, we analyzed single-vehicle crashes involving Hakka and Hoklo drivers, alongside two-vehicle crashes between these groups and Cantonese drivers within Cantonese cultural regions. In this framework, Cantonese drivers in multi-vehicle accidents—who represent the local driving population—serve as the “induced exposure” control group to calibrate the accident odds for non-local cultural groups. While this baseline is theoretically grounded, we acknowledge that systematic differences in trip purpose or vehicle mix (as seen in Table 1) may introduce selection bias. If the control group’s exposure profile deviates significantly from the general population at risk, the resulting Odds Ratios (ORs) should be interpreted as relative risk indicators within this specific regional context. A total of 579 motor vehicle accidents were selected, involving 834 drivers, of which 42.1% were from Hakka cultural regions and 27.1% were from Hoklo cultural regions (Table 1).

**Table 1:** Variable definitions and descriptive statistics.

Variables	Ethnic group			Total N = 834
	Hakka N = 351 (42.1%)	Cantonese N = 257 (30.8%)	Hoklo N = 226 (27.1%)	
Responsibility				
Total/major	207 (59.0%)	85 (33.1%)	120 (53.1%)	412 (49.4%)
Human factor				
(1) Gender				
Male	332 (94.6%)	237 (92.2%)	216 (95.6%)	785 (94.1%)
(2) Age				
Young (<25)	59 (16.8%)	38 (14.8%)	42 (18.6%)	139 (16.7%)
(3) Household registration				
Rural	73 (20.8%)	72 (28.0%)	55 (24.3%)	200 (24.0%)
(4) Occupation				
Staff/civil servant	127 (36.2%)	90 (35.0%)	92 (40.7%)	309 (37.1%)
Worker/farmer	74 (21.1%)	62 (24.1%)	45 (19.9%)	181 (21.7%)
Bosses/self-employed	62 (17.7%)	33 (12.8%)	22 (9.7%)	117 (14.0%)
Otherwise	88 (25.1%)	72 (28.0%)	67 (29.6%)	227 (27.2%)
Vehicle factor				
(5) Ownership of the vehicle				
Commercial	115 (32.8%)	40 (15.6%)	66 (29.2%)	221 (26.5%)
(6) Vehicle type				
Truck	98 (27.9%)	40 (15.6%)	64 (28.3%)	202 (24.2%)
Passenger car	141 (40.2%)	72 (28.0%)	94 (41.6%)	307 (36.8%)
Motorcycle	112 (31.9%)	145 (56.4%)	68 (30.1%)	325 (39.0%)
Road factor				
(7) Road type				
Expressway/urban expressway	73 (20.8%)	36 (14.0%)	30 (13.3%)	139 (16.7%)
Environment factor				
(8) Whether				
Bad	103 (29.3%)	56 (21.8%)	43 (19.0%)	202 (24.2%)
Year				
2006	64 (18.2%)	49 (19.1%)	39 (17.3%)	152 (18.2%)
2007	65 (18.5%)	43 (16.7%)	49 (21.7%)	157 (18.8%)
2008	70 (19.9%)	58 (22.6%)	46 (20.4%)	174 (20.9%)
2009	77 (21.9%)	51 (19.8%)	43 (19.0%)	171 (20.5%)
2010	75 (21.4%)	56 (21.8%)	49 (21.7%)	180 (21.6%)

(1) Hoklo is identified by the Min dialect. (2) Household Registration refers to the Hukou system in China, where “Rural” and “Urban” categories serve as proxies for a driver’s socio-economic background and access to developmental resources. (3) Occupation is classified to reflect professional exposure: “Staff/Civil servants” denote stable institutional employment; “Workers/Farmers” represent manual labor sectors; and “Bosses/Self-employed” refer to individuals in private business or entrepreneurial roles. (4) Age is bifurcated at 25 years to isolate high-risk youth cohorts as defined by World Health Organization (WHO) standards. (5) Expressway/Urban Expressway acts as a proxy for high-grade road infrastructure with controlled access, while Bad Weather aggregates conditions that significantly impair visibility or traction, such as rain, snow, fog, and strong winds.

Past studies have mainly focused on accident casualties (6, 7). However, accident casualties stem from the risk propensity of drivers. For that reason, this study focused on driving behavior and used “primary liability for the accident” as the dependent variable (“1” = primary or full liability and “0” = equal, secondary, or no liability). The control variables included factors related to the driver, vehicle, road, and environment.

Driver characteristics: To control for human factors fundamentally influencing traffic safety, this study incorporated gender, age, and occupation into the analysis. Age is bifurcated into two cohorts—younger than 25 and 25 or older. This threshold is primarily selected because it aligns with World Health Organization (WHO, 29) standards for identifying high-risk youth populations. Moreover, the 25-year cutoff is a widely utilized metric in Chinese traffic safety scholarship to isolate the developmental transition from limited experience to increased driving stability (19). In the context of the current sample, this threshold effectively captures the phase where risk perception and driving behavior are most volatile. Given the absence of direct income or education metrics in official accident records, socio-economic status was operationalized through two indigenous Chinese proxies: Household Registration and occupational category. Household Registration, classified as “Rural” or “Urban,” serves as a critical indicator of a driver’s developmental environment and resource access, while the occupation variable categorizes participants into Staff/Civil servants (representing stable institutional employment), Workers/Farmers (manual labor sectors), Bosses/Self-employed (entrepreneurial/private business), and Others.

Vehicle: As vehicle type significantly influences cross-regional accident risk (25), we categorized involved units into passenger cars, trucks (cargo vehicles), and motorcycles. Furthermore, a binary dummy variable for Commercial Use (1 = commercial; 0 = private) was introduced to isolate the risk patterns inherent to professional transport operations.

Road: Given that infrastructure quality dictates driving patterns, we focused on road classification. China’s road network distinguishes between high-speed infrastructure (Expressways and Urban Expressways) and lower-tier routes (Ordinary and Urban Ordinary roads). We utilized a dummy variable, Expressway/Urban Expressway (1 = high-grade; 0 = other), as a proxy for road quality and driving environment complexity.

Environment: Weather conditions were simplified into a binary variable, Bad Weather. Following Zhang et al. (19), this category aggregates visibility-reducing or traction-limiting conditions, including rain, snow, fog, and strong winds, versus “Clear/Cloudy” conditions.

### 3.2 Descriptive statistics

The characteristics of the study variables are summarized in Table 1. Initial observations indicate that the fault proportions among Hakka and Hoklo drivers were substantially higher than those of their Cantonese counterparts (59.0 and 53.1% vs. 33.1%, respectively). Overall, the sample was predominantly composed of male drivers, individuals aged 25 or older, and those with Urban Household Registration. Regarding occupational distribution, Staff/Civil servants constituted the largest group, followed by Workers/Farmers, while “Bosses”/Self-employed drivers represented the smallest cohort. However, distinct inter-group variations emerged; for instance, the proportion of self-employed Hakka drivers (17.7%) exceeded that of Cantonese (12.8%) and Hoklo drivers (9.7%). In terms of vehicle attributes, Cantonese drivers were significantly less likely to operate commercial vehicles (15.6%) compared to Hakka (32.8%) and Hoklo drivers (29.2%). Furthermore, while the majority of Cantonese drivers operated motorcycles (56.4%), Hakka and Hoklo drivers primarily utilized passenger cars (40.2 and 41.6%, respectively). Regarding road infrastructure, accidents on Expressways/Urban Expressways were more prevalent among Hakka drivers (20.8%) than Cantonese (14.0%) and Hoklo drivers (13.3%). Finally, environmental data showed that the proportion of Hakka drivers involved in accidents during bad weather (29.3%) was notably higher than that of Cantonese (21.8%) and Hoklo drivers (19.0%).

### 3.3 Logistic model

When handling discrete variables, the logistic regression model has an advantage in its ability to measure both the correlation coefficient of the predictors and the direction of the correlation (sign). Due to the increased usage, logistic regression models have become standard for discrete variable correlation, especially in the field of epidemiology (21). Finally, since the dependent variable (responsibility for the accident) was a binary discrete variable, to facilitate comparison with existing studies that had previously adopted a logistic regression model, this study needed to use a logistic regression model for the empirical analysis (22). The logistic regression model function, with the probability that the dependent variable is equal to 1, is expressed as:


$$\mathrm{Pr}[y_{i}=1|x_{i}]=\frac{\exp(x_{i}^{\prime }\beta_{j})}{1+\exp(x_{i}^{\prime }\beta_{j})}$$

Where, $x_{i}$ includes the factors that affect drivers’ risk of traffic accidents, and $0<\mathrm{Pr}[y_{i}=1|x_{i}]<1$ . The coefficient shows the marginal effects of the relative probability of the occurrence of an event. The ratio of the probability of an event occurring to the probability of not occurring is referred to as the odds ratio (*OR*): $\text{OR}=\mathrm{Pr}[y=1]/\mathrm{Pr}[y=0]=\exp(x_{i}^{\prime }\beta_{j})$ . The range for the *OR* was set as [0,∞] , which allows for a measure of the specific change in results caused by the change in the independent variable under the premise that other independent variables remain unchanged, that is, the change ratio of the occurrence the probability of a given variable has to the occurrence probability of the reference level of the variable.

### 3.4 Quantitative comparative analysis

While logistic regression identifies the independent risk of specific factors, it faces limitations when dealing with small sub-group samples or complex interactions between more than three variables (23, 27). Since traffic safety is a complex system where driver, vehicle, and environmental factors rarely act in isolation, we employ Qualitative Comparative Analysis (QCA) as a supplementary method. QCA is uniquely suited for this study because it moves beyond “variable-oriented” analysis to a “configuration-oriented” approach. Instead of asking how much a single factor (like gender) increases risk, QCA asks: “What combination of factors (e.g., a young male driving a motorcycle in bad weather) leads to a high accident risk?”

A key advantage of QCA is its ability to handle asymmetry and multiplicity. In traffic safety, a factor that causes an accident (like being male) does not simply mean its absence (being female) guarantees safety. In reality, female drivers may face higher risks under specific combinations of other conditions. QCA excels at identifying these diverse “paths” to the same outcome. Following the research design of 2), our QCA procedure involves three simplified steps:

1. Necessity and sufficiency analysis: We first test if any single condition is “necessary” (must be present for the accident to occur) or “sufficient” (its presence alone is enough to cause the accident). We use standard thresholds of 0.90 for necessity and 0.85 for sufficiency to filter the most influential factors (28).
2. Truth table construction: We organize all possible combinations of conditions into a “truth table.” Each row represents a specific scenario (e.g., young + male + passenger car). We use Boolean logic to mark the presence (1) or absence (0) of these conditions to see which scenarios consistently result in high-fault odds.
3. Boolean minimization: To simplify the results, we eliminate redundant conditions. If two scenarios have the same outcome but differ by only one factor, that factor is considered irrelevant for that specific path and is removed to find the simplest explanation.

Notably, QCA typically produces three types of solutions: complex, intermediate, and parsimonious. Complex solutions are simplified based strictly on actual case observations. In contrast, intermediate solutions incorporate “easy remainders” supported by theoretical knowledge, while parsimonious solutions include both “easy” and “difficult” remainders to further simplify the results. Following the methodological rationale of Zhang et al. (2), this study reports only complex solutions. This choice is made to ensure the highest level of empirical rigor by avoiding the inherent uncertainty and potential subjective bias associated with the assessment of logical remainders—combinations of conditions that are not present in the actual dataset. By focusing on complex solutions, we ensure that the identified causal paths are grounded entirely in observed evidence, providing a more conservative and reliable interpretation of how cultural backgrounds influence traffic risk.

## 4 Logistic regression analysis

Based on the dependent variable, three independent multivariate logistic regression models were established. The dependent variable was defined as whether the driver had primary liability for the accident. The constructed models included factors related to the driver, vehicle, road, and environment, and risk factors were compared between the Hakka, Cantonese, and Hoklo drivers in Cantonese areas.

The logistic regression results in Table 2 provide detailed insights into the risk factors for each cultural group. Regarding driver characteristics, being younger than 25 is a major risk factor for all groups. This is especially clear for Cantonese drivers, whose Odds Ratio (OR = 2.674) means they are 2.67 times more likely to be at-fault in an accident compared to drivers over 25. For Hakka drivers, Staff/Civil servants show a much higher risk of being at-fault (OR = 3.068), suggesting that drivers in this occupation within the Hakka group may face specific pressures or commuting conditions that affect their safety. In terms of vehicle types, although Cantonese drivers use fewer commercial vehicles, those who do drive them face a very high risk (OR = 2.881). This suggests that Cantonese drivers may be less adapted to the demands of professional driving compared to other groups. On the other hand, Cantonese and Hoklo motorcyclists are much safer than passenger car drivers (OR = 0.234 and 0.349, respectively), which may indicate that riders in these regions are more cautious. Regarding environmental factors, bad weather significantly increases the accident risk for Hakka drivers, while it actually reduces the risk for Cantonese drivers. This difference suggests that Cantonese drivers may adjust their driving speed or caution more effectively when weather conditions worsen, whereas Hakka drivers remain more vulnerable to these changes. These findings show that traffic safety policies should not just focus on general road quality, but should address the specific behaviors and risks of different cultural groups.

**Table 2:** Factors related to driver at-fault crashes.

Variables	(1)	(2)	(3)	
	Hakka	Conton	Hoklo
Human factor			
(1) Gender			
Male	1.279	2.609	1.553
	[0.464, 3.525]	[0.728, 9.346]	[0.389, 6.199]
(2) Age			
Young (<25)	**1.745** ^*^	**2.674** ^**^	**1.940** ^*^
	**[0.932, 3.265]**	**[1.119, 6.394]**	**[0.904, 4.162]**
(3) Household registration			
Rural	0.892	0.865	0.598
	[0.502, 1.582]	[0.429, 1.744]	[0.300, 1.190]
(4) Occupation (relative to staff/civil servant)			
Worker/farmer	**3.068** ^***^	1.349	1.546
	**[1.470, 6.405]**	[0.494, 3.687]	[0.499, 4.789]
Boss	1.757	1.477	1.500
	[0.894, 3.454]	[0.527, 4.143]	[0.539, 4.176]
Otherwise	1.832^*^	0.547	2.165
	[0.909, 3.693]	[0.195, 1.535]	[0.746, 6.278]
Vehicle factor			
(5) Ownership of the vehicle			
Commercial	0.840	**2.881** ^**^	1.150
	[0.478, 1.476]	**[1.152, 7.208]**	[0.507, 2.609]
(6)Vehicle type (relative to passenger car)			
Truck	0.807	0.507	0.850
	[0.439, 1.482]	[0.197, 1.304]	[0.369, 1.962]
Motor cycle	0.981	**0.234** ^***^	**0.349** ^***^
	[0.551, 1.747]	**[0.109, 0.503]**	**[0.167, 0.729]**
Road factor			
(7) Road type			
Expressway/urban expressway	1.446	0.842	0.701
	[0.806, 2.595]	[0.355, 2.000]	[0.303, 1.626]
Environment factor			
(8) Whether			
Bad	**1.526** ^*^	**0.352** ^***^	1.464
	**[0.924, 2.520]**	**[0.160, 0.774]**	[0.694, 3.087]
Year (relative to 2006)			
2007	3.537^***^	1.574	1.906
	[1.655, 7.558]	[0.582, 4.262]	[0.765, 4.750]
2008	1.782	1.993	1.073
	[0.869, 3.651]	[0.786, 5.053]	[0.422, 2.729]
2009	1.828^*^	2.514^*^	1.125
	[0.910, 3.673]	[0.967, 6.533]	[0.436, 2.904]
2010	2.160^**^	1.997	1.534
	[1.054, 4.424]	[0.760, 5.245]	[0.621, 3.787]
pseudo *R*^2^	0.062	0.128	0.078
r2_cu	0.108	0.209	0.137
ll	−222.944	−142.195	−144.029
chi2	29.331	41.847	24.377
lrx2_p	0.015	0.000	0.059
aic	477.889	316.389	320.057
bic	539.661	373.174	374.786
pcorrectly	63.533	73.541	64.602
ROC	0.663	0.734	0.693
*N*	351	257	226

The above results are estimated by Stata 17.

Exponentiated coefficients; 95% confidence intervals in brackets.

^*^p < 0.1, ^**^p < 0.05, ^***^p < 0.01.Bold text denotes the coefficients of significant variables.

The above findings showed that the propensity toward risky driving behaviors was different between ethnic groups. To further verify the differences, this study drew on the method proposed by 24) to test the structural differences among the three models. The results of the Chow test showed that $\chi^{2}$ was 80.26 when the degrees of freedom were set as 32, and the null hypothesis that there were no structural differences between the three models was rejected at the 1% significance level. Therefore, drivers’ driving behaviors from the three groups can be considered significantly different.

We find that certain variables in the logistic regression, such as gender, exhibit wide confidence intervals. This instability often occurs in traditional regression when specific sub-groups (e.g., female drivers involved in accidents in non-local cultural areas) have a relatively small sample size, or when complex interactions between variables are not fully captured by a linear model. To address this limitation and provide a more robust analysis, we further employ Qualitative Comparative Analysis (QCA). Unlike logistic regression, which estimates the independent effect of a single variable, QCA is designed to identify “configurations” of multiple factors that lead to a specific outcome. This approach is particularly suitable for our study as it does not rely on large sample sizes for every individual variable and can reveal how different combinations of driver, vehicle, and environment factors together influence traffic risk. By moving from a variable-oriented to a configuration-oriented analysis, we can better understand the complex safety patterns that a traditional model might miss.

## 5 Qualitative comparative analysis

The logistic regression analysis results showed that gender, household registration, some occupations, cargo vehicle, and road type had no significant impact on behaviors that caused traffic accidents or serious casualties for either group. Further investigation was needed in terms of the influence these variables had on traffic safety outcomes. This section showed the results of the QCA to analyze risk-taking driving behaviors of Hakka and Hoklo drivers and how factors related to driver, vehicle, road, and environment interact with one another to affect traffic accident risk.

Based on this study’s needs, four cs-QCA were conducted. The first two analyses targeted Hakka drivers, with the dichotomous “primary liability for the accident” variable used as the outcome variable and driver, vehicle, road, and environmental factors as antecedent conditions. Independent calculations were performed according to the two outcomes (“1” = traffic accident and “0” = no traffic accident) to analyze the combination of variables that lead to the occurrence/non-occurrence of a traffic accident. The latter two analyses were an independent investigation of Hoklo drivers. It should be noted that according to the Boolean minimization analysis, there are generally three kinds of solutions reported in the QCA analysis results: complex solutions, intermediate solutions, and parsimonious solutions. Complex solutions are combined conditions, simplified based on actual observation. Intermediate solutions both analyze actual observations and incorporate “easy remainders” supported by theoretical and/or practical knowledge to simplify the expression. Parsimonious solutions use actual observations as well as “easy and difficult remainders” (48). Considering the uncertainty of the determination of the logical remainder, only complex solutions were reported.

### 5.1 Hakka drivers’ behavioral patterns

Table 3 shows that none of the variables met the threshold for either sufficient or necessary condition, suggesting that there was no single sufficient and necessary condition that caused Hakka drivers’ propensity to traffic accidents in Cantonese areas, that is, the individual condition had weak explanatory power. Therefore, it was necessary to conduct a subsequent analysis combining the conditions.

**Table 3:** Necessity and sufficiency of the conditions of Hakka drivers in Canton: outcome variable of “1” (there is a driver at-fault crash).

Variables	Total/major		Not total/major		
	Consistency	Coverage	Consistency	Coverage
Male	0.9469	0.5904	0.9444	0.4096
Not male	0.0531	0.5789	0.0556	0.4211
Young	0.1981	0.6949	0.1250	0.3051
Not young	0.8019	0.5685	0.8750	0.4315
Rural	0.2029	0.5753	0.2153	0.4247
Not rural	0.7971	0.5935	0.7847	0.4065
Staff/civil servant	0.1401	0.4677	0.2292	0.5323
Not staff/civil servant	0.8599	0.6159	0.7708	0.3841
Boss	0.2464	0.6892	0.1597	0.3108
Not boss	0.7536	0.5632	0.8403	0.4368
Worker/farmer	0.3575	0.5827	0.3681	0.4173
Not worker/farmer	0.6425	0.5938	0.6319	0.4063
Commercial	0.3043	0.5478	0.3611	0.4522
Not commercial	0.6957	0.6102	0.6389	0.3898
Truck	0.2609	0.5510	0.3056	0.4490
Not truck	0.7391	0.6047	0.6944	0.3953
Passenger car	0.4058	0.5957	0.3958	0.4043
Not passenger car	0.5942	0.5857	0.6042	0.4143
Motor cycle	0.3333	0.6161	0.2986	0.3839
Not motor cycle	0.6667	0.5774	0.7014	0.4226
Expressway/urban expressway	0.2367	0.6712	0.1667	0.3288
Not expressway/urban expressway	0.7633	0.5683	0.8333	0.4317
Bad weather	0.3237	0.6505	0.2500	0.3495
Not bad weather	0.6763	0.5645	0.7500	0.4355

The above results are estimated by fs/QCA 3.0.

According to the results computed using the fs/QCA software, 41 complex solution combinations leading to traffic accidents (value = “1”) were obtained, with a total coverage of 0.527 and consistency of 0.924. When the value of the outcome was “0” (no traffic accident), 31 complex solution combinations were obtained, with a total coverage of 0.354 and a consistency of 0.944.

The results demonstrated the complexity of the formation of traffic accidents that involved drivers from other cultures, as no single factor could explain such a formation. In addition, these findings verified the asymmetry and multiplicity in the relationship between Hakka drivers’ involvement in traffic accidents in areas of Cantonese culture and corresponding risk factors. For example, consider gender, although there were more configurations containing “male” as a condition than that containing “female” as a condition (33 vs. 5), gender was not the only condition that differed. Such a discrepancy suggested that the relationship between Hakka drivers’ gender and their involvement in traffic accidents had a multiplicity effect. Moreover, the configurations with “male” as a condition for “traffic accident” could not be derived into configurations of “no traffic accident” with “female” as a condition. These findings indicated that male Hakka drivers’ higher risk of road traffic accidents did not suggest fewer accidents for female drivers. Therefore, the relationship between Hakka drivers’ gender and their involvement in traffic accidents was asymmetric.

Comparing the solution configurations for “traffic accident” as an outcome and that of “no traffic accident” as the outcome might provide additional information on the interactions between driver, vehicle, road, and environment and how such interactions affect the occurrence of road traffic accidents. The results showed that, in specific scenarios, changes in a single condition were key to the difference in outcome (see Table 4). In Scenario 1, among young, male Hakka drivers with an urban household registration that drove non-commercial cargo vehicles on roads that were not expressways/urban expressways, self-employed workers had a higher risk than workers/farmers. However, Scenario 2 shows that workers/farmers driving commercial cargo vehicles had a higher risk than those driving non-commercial cargo vehicles. In Scenario 3, among older, male Hakka drivers with a rural household registration that drove non-commercial passenger vehicles on roads that were not expressways/urban expressways, workers/farmers had a higher risk than clerks/civil servants. In Scenario 4, among male Hakka drivers with an urban household registration that drove non-commercial cargo vehicles on roads that were not expressways/urban expressways in good weather, young drivers had a higher risk of traffic accidents. However, Scenario 5 shows that older drivers that drove commercial cargo vehicles had a higher risk than those driving non-commercial cargo vehicles. In Scenario 6, it was found that, among older, self-employed Hakka drivers that drove commercial motorcycles on roads that were not expressways/urban expressways in good weather, females had a higher risk of accident than males.

**Table 4:** Causal paths toward crash liability of drivers from Hakka driving in Canton.

Variables	Scenario 1		Scenario 2		Scenario 3		Scenario 4		Scenario 5		Scenario 6		
	Yes	No	Yes	No	Yes	No	Yes	No	Yes	No	Yes	No
Male	●	●	●	●	●	●	●	●	●	●	○	●
Yuang	●	●	●	●	○	○	●	○	○	○	○	○
Rural household registration	○	○	○	○	●	●	○	○	○	○	○	○
Staff	○	○	○	○	○	●	●	●	●	●	○	○
Boss	●	○	○	○	○	○	○	○	○	○	●	●
Worker/farmer	○	●	●	●	●	○	○	○	○	○	○	○
Commercial	○	○	●	○	○	○	○	○	●	○	●	●
Trunk	●	●	●	●	○	○	●	●	●	●	○	○
passenger car	○	○	○	○	●	●	○	○	○	○	○	○
Motorcycle	○	○	○	○	○	○	○	○	○	○	●	●
Expressway	○	○	○	○	○	○	○	○	○	○	○	○
Bad weather							○	○	○	○	○	○
Unique coverage	0.0097	0.0069	0.0048	0.0069	0.0145	0.0069	0.0048	0.0208	0.0145	0.0208	0.0048	0.0069

The above results are estimated by fs/QCA 3.0.

● indicates that the variable appears, ○ indicates that the variable does not appear, _ indicates a difference.

### 5.2 Hoklo drivers’ behavioral patterns

Similarly, as can be found in Table 5, none of the variables met the threshold for sufficient or necessary conditions, suggesting that there was no single sufficient and necessary condition for Hoklo drivers to cause traffic accidents in Cantonese areas, that is, the individual conditions also had weak explanatory power, and it was also necessary to conduct a combined analysis.

**Table 5:** Necessity and sufficiency of the conditions of Hoklo drivers in Canton: Outcome variable of “1” (there is a driver at-fault crash).

Variables	Total/major		Not total/major		
	Consistency	Coverage	Consistency	Coverage
Male	0.9667	0.5370	0.9434	0.4630
Not male	0.0333	0.4000	0.0566	0.6000
Young	0.2167	0.6190	0.1509	0.3810
Not young	0.7833	0.5109	0.8491	0.4891
Rural	0.1917	0.4182	0.3019	0.5818
Not rural	0.8083	0.5673	0.6981	0.4327
Staff/civil servant	0.1000	0.5455	0.0943	0.4545
Not staff/civil servant	0.9000	0.5294	0.9057	0.4706
Boss	0.1917	0.5111	0.2075	0.4889
Not boss	0.8083	0.5359	0.7925	0.4641
Worker/farmer	0.3833	0.5000	0.4340	0.5000
Not worker/farmer	0.6167	0.5522	0.5660	0.4478
Commercial	0.3333	0.6061	0.2453	0.3939
Not commercial	0.6667	0.5000	0.7547	0.5000
Truck	0.3167	0.5938	0.2453	0.4063
Not truck	0.6833	0.5062	0.7547	0.4938
Passenger car	0.4750	0.6064	0.3491	0.3936
Not passenger car	0.5250	0.4773	0.6509	0.5227
Motor cycle	0.2083	0.3676	0.4057	0.6324
Not motor cycle	0.7917	0.6013	0.5943	0.3987
Expressway/urban expressway	0.1083	0.4333	0.1604	0.5667
Not expressway/urban expressway	0.8917	0.5459	0.8396	0.4541
Bad weather	0.2250	0.6279	0.1509	0.3721
Not bad weather	0.7750	0.5082	0.8491	0.4918

The above results are estimated by fs/QCA 3.0.

The results of the QCA of Hoklo drivers also showed asymmetric and multiplicity in the relationship between driving in areas of another culture and involvement in traffic accidents. Specifically, 29 complex solution combinations leading to an outcome of traffic accident (value = “1”) were obtained, with a total coverage of 0.567 and consistency of 0.971. When the value of the outcome was “0” (no traffic accident), 24 complex solution combinations were obtained, with a total coverage of 0.443 and a consistency of 0.979.

As with the findings of the Hakka drivers, in specific scenarios, changes in a single condition were key to the differences in the outcomes for Hoklo drivers (see Table 6). In Scenario 1, among older, male, self-employed Hoklo drivers that drove non-commercial cargo vehicles on roads that were not expressways/urban expressways in good weather, drivers with a rural household registration had a higher risk than those with urban household registration. In Scenario 2, among older, male Hoklo drivers with a rural household registration that drove on roads that were not expressways/urban expressways in good weather, drivers of commercial passenger vehicles had a higher risk than those of non-commercial passenger vehicles. In Scenario 3, for older Hoklo clerks/civil servants with urban household registration that drove commercial passenger vehicles on expressways/urban expressways, the traffic accident risk was greater during bad weather. In Scenario 4, for older, Hoklo workers/farmers with urban household registration that drove non-commercial cargo vehicles during bad weather, the traffic accident risk was greater when they were on expressways/urban expressways. In Scenario 5, for older, male, self-employed Hoklo drivers with urban household registration that drove commercial cargo vehicles in bad weather, the traffic accident risk was greater when they were on expressways/urban expressways. In addition, according to Scenario 6, when driving on expressways/urban expressways, their traffic accident risk was greater during good weather.

**Table 6:** Causal paths toward crash liability of drivers from Hoklo driving in Canton.

Variables	Scenario 1		Scenario 2		Scenario 3		Scenario 4		Scenario 5		Scenario 6		
	Yes	No	Yes	No	Yes	No	Yes	No	Yes	No	Yes	No
Male	●	●	●	●	●	●	●	●	●	●	●	●
Yuang	○	○	○	○	○	○	○	○	○	○	○	○
Rural household registration	●	○	●	●	○	○	○	○	○	○	○	○
Staff	○	○	●	●	●	●	○	○	○	○	○	○
Boss	●	●	○	○	○	○	○	○	●	●	●	●
Worker/farmer	○	○	○	○	○	○	●	●	○	○	○	○
Commercial	○	○	●	○	●	●	○	○	●	●	●	●
Trunk	●	●	○	○	○	○	●	●	●	●	●	●
Passenger car	○	○	●	●	●	●	○	○	○	○	○	○
Motorcycle	○	○	○	○	○	○	○	○	○	○	○	○
Expressway	○	○	○	○	○	○	●	○	○	●	●	●
Bad weather	○	○	○	○	●	○	●	●	●	●	○	●
Unique coverage	0.0167	0.0094	0.0083	0.0094	0.0083	0.0094	0.0083	0.0094	0.0167	0.0094	0.0083	0.0094

The above results are estimated by fs/QCA 3.0.

● indicates that the variable appears, ○ indicates that the variable does not appear, _ indicates a difference.

## 6 Discussion

The empirical results reveal that traffic risk factors are not uniform but are significantly modulated by the sub-cultural backgrounds of drivers within a unified administrative and legal environment. While traditional regression identifies age as a universal risk factor, the more nuanced cultural variations in response to weather, occupation, and vehicle types suggest that driving behavior is a manifestation of regional social norms, long-term environmental conditioning, and ingrained cognitive schemata.

### 6.1 Geographic-cultural adaptation and the climate-cognition gap

A comparative analysis of these findings against existing literature reveals a “climate-culture cognition gap” where geographic origins act as a primary moderator of traffic risk. First, as summarized in the comparative synthesis of Table 7, weather conditions often show no significant independent impact in the United States or the Hong Kong-Macao cross-border study (2). In contrast, our study found that bad weather significantly increased risk for Hakka drivers while acting as a protective factor for Cantonese drivers. From a theoretical perspective, this reflects a “geographic-cultural adaptation” where Cantonese and Hoklo “marine cultures” have developed a robust risk compensation mechanism due to their historical familiarity with coastal storms (19). The Hakka “mountain culture,” however, originates from inland regions far from the ocean, leading to a lack of ingrained cognitive resilience to coastal weather patterns and resulting in higher fault odds when faced with environmental volatility. Second, Table 7 highlights a discrepancy regarding gender effects; while 2, 19) identified male gender as a dominant risk factor, our results suggest that within a shared legal framework, cultural affinity can neutralize gender-based behavioral traits. This indicates that the higher risk for male drivers in previous literature may be driven more by institutional friction, such as changing traffic directions, than by biological gender alone.

**Table 7:** Comparison of results with previous studies.

Variables	Mainland China (19)	Cross-border from Hong Kong and Macau in Mainland China (2)	Cross-state in USA (Harootunian et al.)
Human factor			
(1) Gender	Male (+)	Male (+)	Male(+)
(2) Age	Not significant	–	–
(3) Household registration	Rural (+)	–	–
(4) Occupation	Not significant	–	–
Vehicle factor			
(5) Ownership of the vehicle	Not significant	Not significant	–
(6) Vehicle type	Motorcycle (−)		–
Road factor			
(7) Road type	–	Not significant	Not significant
Environment factor			
(8) Whether	Bad (−)	Not significant	–

### 6.2 Configurational nature of risk: moving beyond independent effects

The QCA results provide a necessary theoretical expansion of the “variable-oriented” approach by demonstrating that accident risk is a configurational outcome driven by social positioning and group norms. First, although the logit model and previous studies in Table 7 suggested gender and Household Registration were often independently insignificant, QCA uncovered that socio-economic status operates through “cultural resource-access clusters” (49, 50). For example, the high-risk configuration of self-employed Hakka drivers reflects the “entrepreneurial pressure” found in certain sub-cultures, where the drive for economic efficiency may lead to a cultural normalization of aggressive driving or fatigue. Second, vehicle types reflect “cultural role adaptation”; the significantly lower fault odds for Cantonese motorcyclists, which is consistent with the direction of effect in 19) as noted in Table 7, suggest a localized survival awareness where riders have adapted through compensatory caution. Third, the indefinite impact of road type observed in the QCA results supports “Risk Homeostasis Theory,” indicating that the safety benefits of better infrastructure are mediated by cultural perception (19). Specifically, certain groups, such as male Hakka workers on high-grade expressways, may perceive a higher safety margin and subsequently increase their speed, leading to a higher accident probability despite superior road quality.

### 6.3 Limitations

A primary conceptual challenge in this study is the potential confounding of cultural norms with regional environmental exposure. While we utilize dialect as a sophisticated proxy for cultural lineage—which offers higher granularity than nationality—dialect regions in Guangdong remain geographically clustered. Consequently, our findings may partially reflect residual regional effects, such as varying levels of urbanization, terrain-specific driving familiarity, or subtle differences in local law enforcement intensity. However, we contend that the observed behavioral variations represent more than mere environmental adaptation. From an evolutionary perspective, cultural norms and regional adaptations are inextricably linked; long-term exposure to specific ecological and institutional constraints often crystallizes into persistent cultural traits and economic preferences. As noted by (43), such cultural footprints exhibit long-term continuity and are resistant to short-term institutional shifts. Furthermore, global evidence suggests that these ingrained preferences (e.g., risk appetite) directly translate into varied propensities for risk-taking behavior across different populations. By conducting this research within a unified institutional framework in Guangdong, we effectively neutralize the confounding effects of disparate traffic laws and national infrastructures. This approach aligns with the methodology of (2), who demonstrated that even within shared legal environments, cultural determinants remain a primary driver of risky behavior. Thus, the documented disparities between Cantonese, Hakka, and Hoklo drivers likely reflect deeply ingrained cultural ‘behavioral scripts’ that transcend immediate geographic boundaries.

## 7 Conclusion

This study systematically identifies the structural differences in driving behavior across Hakka, Cantonese, and Hoklo cultural groups by utilizing Chinese dialects as a proxy for ethnic cultural backgrounds. First, while traditional logistic regression highlights universal risk factors such as young age, the Qualitative Comparative Analysis (QCA) reveals that accident risk is a configurational outcome where individual, vehicle, and environmental factors converge. Specifically, factors like gender, Household Registration, and occupation—which may appear insignificant in isolation—become critical when combined in certain paths, such as the high-risk clusters identified for self-employed Hakka drivers or rural male truck operators. Second, unlike research on transnational or cross-border driving where language barriers and legal discrepancies often confound results (2,12), the differences observed here are primarily rooted in sub-cultural identity and long-term environmental conditioning.

These findings provide a critical foundation for transitioning from general traffic management to “precision governance” through culturally-aligned regulations. First, our findings argue against “one-size-fits-all” safety policies; instead, authorities should develop specialized training programs that address the specific cognitive gaps identified, such as mandating bad-weather driving education for drivers from inland mountain-culture backgrounds (Hakka) when they relocate to coastal regions. Second, the “indeterminacy” of road quality in our results, which aligns with the Risk Homeostasis Theory (19), suggests that regulation should focus on “configurational monitoring” rather than just infrastructure upgrades. Law enforcement should be concentrated on high-risk clusters where multiple factors—such as specific occupations, vehicle types, and timing—converge. Third, for commercial vehicle regulation, the high risk observed among specific cultural cohorts suggests that a more granular licensing or monitoring system for special-purpose vehicles, as suggested in related regional studies (2), could be an effective method to mitigate culturally-specific risk patterns.

It is worth noting that although the QCA model has overcome some of the inherent limitations of the logistic model, the QCA model has its own limitations, such as inability to cope with missing data or data with poor quality, incorrect encoding process and data specification (2). Due to the limitation of the number of data samples, this paper did not incorporate more refined vehicle, road or environmental factors into the model. Although this paper verified that different ethnic cultures can lead to different risk behavior patterns, it failed to deeply explore the reasons and paths by which different ethnic cultures lead to different behavior patterns from the perspective of economic theory. Therefore, in future research, we will expand the study on the influence of culture on risk behavior based on economic risk preference and risk behavior theory, explore the potential influence of other factors, and consider more detailed variable division and driving behavior models of more ethnic-cultural groups.

## Data Availability

The datasets presented in this article are not readily available because the traffic accident data that support the findings of this study are available from Ministry of Public Security (China), but restrictions apply to the availability of these data, which were used under licence for the current study and so are not publicly available. Requests to access the datasets should be directed to https://www.mps.gov.cn/.
